# Effect of ultrafiltration on extravascular lung water assessed by lung ultrasound in children undergoing cardiac surgery: a randomized prospective study

**DOI:** 10.1186/s12871-019-0771-1

**Published:** 2019-06-04

**Authors:** Mohamed Elayashy, Mai A. Madkour, Ahmed Abdelaal Ahmed Mahmoud, Hisham Hosny, Amr Hussein, Ahmed Nabih, Ahmed Lofty, Hamza Mohamed Hamza, Passaint Hassan, Mohamed Wagih, Ahmed Kareem Mohamed

**Affiliations:** 1 Department of Anesthesia and Intensive Care, Kasr Al Ainy Faculty of Medicine, Cairo University, 7 Elshishiny St., El Maryotia, Faysal, Giza, 12131 Egypt; 2 Department of Anesthesia, Faculty of Medicine, Beni-Suef University, Beni-Suef, Egypt

**Keywords:** Lung ultrasound, Ultrafiltration, Paediatric cardiac surgery, Extravascular lung water

## Abstract

**Background:**

Increased lung water and the resultant atelectasis are significant pulmonary complications after cardiopulmonary bypass (CPB) in children undergoing cardiac surgery; these complications are observed after CPB than after anaesthesia alone. Ultrafiltration has been shown to decrease total body water and postoperative blood loss and improve the alveolar to arterial oxygen gradient and pulmonary compliance. This study investigated whether conventional ultrafiltration during CPB in paediatric heart surgeries influences post-bypass extravascular lung water (EVLW) assessed by lung ultrasound (LUS).

**Methods:**

This randomized controlled study included 60 patients with congenital heart disease (ASA II-III), aged 1 to 48 months, with a body weight > 3 kg. Conventional ultrafiltration targeting a haematocrit (HCT) level of 28% was performed on the ultrafiltration group, while the control group did not receive ultrafiltration. LUS scores were recorded at baseline and at the end of surgery. The PaO2/FiO2 ratio (arterial oxygen tension divided by the fraction of inspired oxygen), urine output, and haemodynamic parameters were also recorded.

**Results:**

LUS scores were comparable between the two groups both at baseline (*p* = 0.92) and at the end of surgery (*p* = 0.95); however, within the same group, the scores at the end of surgery significantly differed from their baseline values in both the ultrafiltration (*p* = 0.01) and non-ultrafiltration groups (*p* = 0.02).

The baseline PaO2/FiO2 ratio was comparable between both groups. at the end of surgery, The PaO2/FiO2 ratio increased in the ultrafiltration group compared to that in the non-ultrafiltration group, albeit insignificant (*p* = 0.16). no correlation between the PaO2/FiO2 ratio and LUS score was found at baseline (r = − 0.21, *p* = 0.31). On the other hand, post-surgical measurements were negatively correlated (r = − 0.41, *p* = 0.045).

**Conclusion:**

Conventional ultrafiltration did not alter the EVLW when assessed by LUS and oxygenation state. Similarly, ultrafiltration did not affect the urea and creatinine levels, intensive care unit (ICU) stays, ventilation days, or mortality.

**Trial registration:**

Clinicaltrials.gov Identifier: NCT03146143 registered on 29-April-2017.

## Background

In children, increased lung water and the resultant atelectasis are significant pulmonary complications after cardiac surgery with cardiopulmonary bypass (CPB). These complications are observed more after CPB than after anaesthesia alone [1]. interstitial oedema may lead to changes in the intrinsic elastic properties of the lung parenchyma during CPB. In combination with the action of constrictor mediators, oedema produces an obstructive process in the bronchi leading to both atelectasis and bronchospasm [2]. These pulmonary complications are usually associated with low arterial oxygen pressure (PaO2) or high carbon dioxide pressure (PaCO2), which may continue for several days, leading to prolonged mechanical ventilation [3].

CPB is also responsible for the activation of leukocytes and inflammatory processes resulting in alteration of capillary permeability as well as interstitial oedema [4]. Paediatric patients are more sensitive to factors like anticoagulation, haemodilution, hypothermia and the exposure of blood to non-endothelialised surfaces. Which initiate a systemic inflammatory response that increases the total body water and extravascular lung water (EVLW) [5].

Ultrafiltration targets the removal of inflammatory mediators throughout CPB together with modest haemoconcentration without prolonging the CPB [6]. Ultrafiltration has been shown to decrease total body water and postoperative blood loss and also to improve alveolar-to-arterial oxygen gradient and pulmonary compliance [7]. Compared to adults, children benefit more from ultrafiltration as they have an exaggerated inflammatory response from an increased bypass-surface to blood-volume ratio, higher exposure to blood transfusion, and less developed immune system [8]. This hypothesis is supported by a more pronounced suppression of inflammatory mediators by ultrafiltration in children compared to adults [9].

Lung ultrasound (LUS) can identify the most common pathologic abnormalities of the respiratory system encountered with high diagnostic accuracy including pneumothorax, pleural effusions, consolidation, and interstitial syndrome, which significantly affect patient management [10]. LUS also has a high diagnostic accuracy for identifying fluid overload and increased lung water and can therefore replace chest X-ray in paediatric patients [11].

In the current research we studied the effect of ultrafiltration during CPB on post-bypass EVLW using LUS and its effect on oxygenation. To the best of our knowledge, no previous study has assessed the effect of conventional ultrafiltration during CPB on EVLW by using bedside LUS in children undergoing cardiac surgery.

## Methods

This is a single-centre, open-label, randomized controlled study conducted at Cairo University Paediatric Hospital after obtaining institutional Research Ethics Committee approval and written informed consent from patients’ guardians. Patients were enrolled from 15th of May 2017 until 5th of October 2017. The study is also registered at ClinicalTrials.gov (NCT03146143).

Included in the study were 60 patients with congenital heart disease (ASA II-III), age ranged from 1 to 48 months, and with body weight > 3 kg. Children with lung disease (asthma, bronchiectasis), those who were mechanically ventilated preoperatively, those with renal/hepatic impairment (values more than double the upper reference range), or those on preoperative inotropic support were excluded. a sequence generated by computer randomly allocated patients into ultrafiltration group (*n* = 30) and non-filtration (control) group (*n* = 30). Sealed opaque envelopes were used for concealment.

Preoperative assessment was performed according to our institutional protocol. Medication history revealed the use of thiazide diuretics as chronic medication to relieve pulmonary volume overload. Patients received intramuscular midazolam 0.3 mg/kg and atropine 0.02 mg/kg as pre-medication 15 min before induction.

Induction of anaesthesia was established using fentanyl 1–5 μg/kg and ketamine 1–2 mg/kg. Atracurium 0.5 mg/kg facilitated endotracheal intubation and maintained intraoperative muscle relaxation as needed. Anaesthesia was maintained by expired sevoflurane 0.3–2% in the oxygen-air mixture (1:1 flow ratio) to obtain an FiO2 of 60%. Ventilation was adjusted in volume-controlled mode with a respiratory rate targeting PaCO2 between 35 and 40 mmHg. The tidal volume was maintained at 8 to 10 mL/kg, and the positive end-expiratory pressure (PEEP) was 4 cmH2O. The inspiratory-to-expiratory ratio was 1:2. A central venous catheter (Arrow International Inc., Reading, PA, USA) was inserted, and an arterial line was used for invasive blood pressure monitoring. On CPB, anaesthesia was maintained with sevoflurane and midazolam 0.1 mg/kg/h infusion.

In all patients, a median sternotomy was performed. CPB was initiated after full heparinization at a dose of 300–400 IU/kg to achieve an ACT of 450 s and standard aorta-bicaval cannulation. A membrane oxygenator (Minimax Plus; Medtronic, Inc., Anaheim, CA) and a non-pulsatile roller pump (model 10.10.00; Stôckert Instruments; Munich, Germany) were used. A small incision at the interatrial septum facilitated the insertion of the left atrial vent to vent the left heart. Priming fluids consisted of lactated Ringer’s solution supplemented with heparin. Blood was added to the priming solution to achieve a haematocrit (HCT) of 28% at the start of CPB. Moderate hypothermia (28 °C) was used during CPB. The aorta was completely cross-clamped, and myocardial preservation was achieved via antegrade cold enriched blood cardioplegia at a dose of 40 mL/kg. Furosemide 1 mg//kg was given after initiation of the bypass. Conventional ultrafiltration was performed after placement of the haemoconcentrator D575 with DHF02 (Sorin Group Italia, s.r.l., LIVANOVA) with its inlet connected to the arterial line and outlet to the cardiotomy or to the venous reservoir. After the initiation of CPB and stabilization of the haemodynamics according to the standardized parameters, hemofiltration was started and continued for up to 10 min before weaning from CPB to maintain the HCT value at 28%.

After surgical repair, de-airing, rewarming and aortic declamping were performed, and the lungs were recruited with a continuous positive airway pressure (C-PAP) of 30 cmH2O for 40 s. Then, the pre-bypass mechanical ventilation mode was resumed. The use of inotropes was guided by the patient’s haemodynamic parameters. Dobutamine 5–10 μg/kg/min, nitroglycerin 1–4 μg/kg/min, adrenaline 0.01–0.05 μg/kg/min or milrinone 0.2–0.5 μg/kg/min were used according to the pathophysiology and the intraoperative state of the patient. Heparin was reversed with protamine sulphate at a ratio of 1 mg per 100 IU of heparin.

The ultrafiltration group was subjected to conventional ultrafiltration targeting HCT level of 28% monitored by arterial blood gases, while the control group (non-filtration group) had no ultrafiltration and blood was transfused to target the same HCT level.

### Assessment of EVLW using LUS

LUS was performed to diagnose EVLW. An M-Turbo SonoSite ultrasound system with a paediatric linear probe (frequency 13–6 MHz; Fujifilm, SonoSite, Inc., USA) was used. Chest ultrasound was performed using the twelve-region method. Intercostal spaces on each side were examined anteriorly (midclavicular line), laterally (anterior axillary line) and posteriorly (posterior axillary line) [12].

Scores of 0 to 3 were given for each region [13].

- Score 0: Normal aeration (N); standard sliding with A-lines or less than 3 B-lines;
- Score 1: Moderate loss of lung aeration; multiple visible B-lines with horizontal spacing between adjacent B-lines ≤7 mm (B7 lines);
- Score 2: Severe loss of lung aeration; multiple B-lines fused together with horizontal spacing between adjacent B; lines ≤3 mm (B3 lines); and
- Score 3: Pulmonary consolidation; hypoechoic lung tissue with dynamic air bronchogram.

The final LUS score of the patient was the sum of each regional ultrasound score ranging from 0 to 36. The LUS score was recorded at baseline 5 min after induction of anaesthesia and at the end surgery after skin closure.

The primary endpoint of the current study was the LUS score at the end of surgery. The PaO2/FiO2 ratio (arterial oxygen tension divided by the fraction of inspired oxygen) and haemodynamic parameters (heart rate, blood pressure) at baseline and at the end of surgery were also recorded. The volume of ultrafiltrate and urine output were also recorded at the end of surgery and day 1 (D1) postoperatively. Urea and creatinine were recorded at baseline and D1 in the intensive care unit (ICU). Additionally, the duration of mechanical ventilation, mortality and patient characteristics were recorded.

### Statistical analysis

Our main primary outcome (LUS score) was presented as the median (IQR), and haemodynamic parameters were presented as the mean (SD). A general linear model was performed for between-group comparisons of lung scores and the PaO2/FiO2 ratio, and the same model with age adjustment as the covariate was applied for paired analysis of the same group variables. Unpaired and paired Student t-tests were used to compare haemodynamics. Categorical data are presented as frequencies and were compared by using the chi square test. Correlations between the PaO2/FiO2 ratio and LUS score were determined using the Pearson moment correlation equation. A *p* value < 0.05 was used as the level to determine statistically significant differences. All statistical calculations were performed using the SPSS version 23 (Statistical Package for the Social Science; SPSS, Inc., Chicago, IL, USA) statistical program. Data for 10 patients from our centre showed that the mean ± SD of the lung score of these patients subjected to non-ultrafiltration was 13.1 ± 5. With the assumption of a 30% difference between groups and by using G power software (version 3.1.3, Heinrich-Heine-Universität, Düsseldorf Germany) with power of 0.8 and 0.05 alpha error, the sample size was 27 per group, rolled up to 30 to allow for possible dropouts.

## Results

**Fig. 1:**
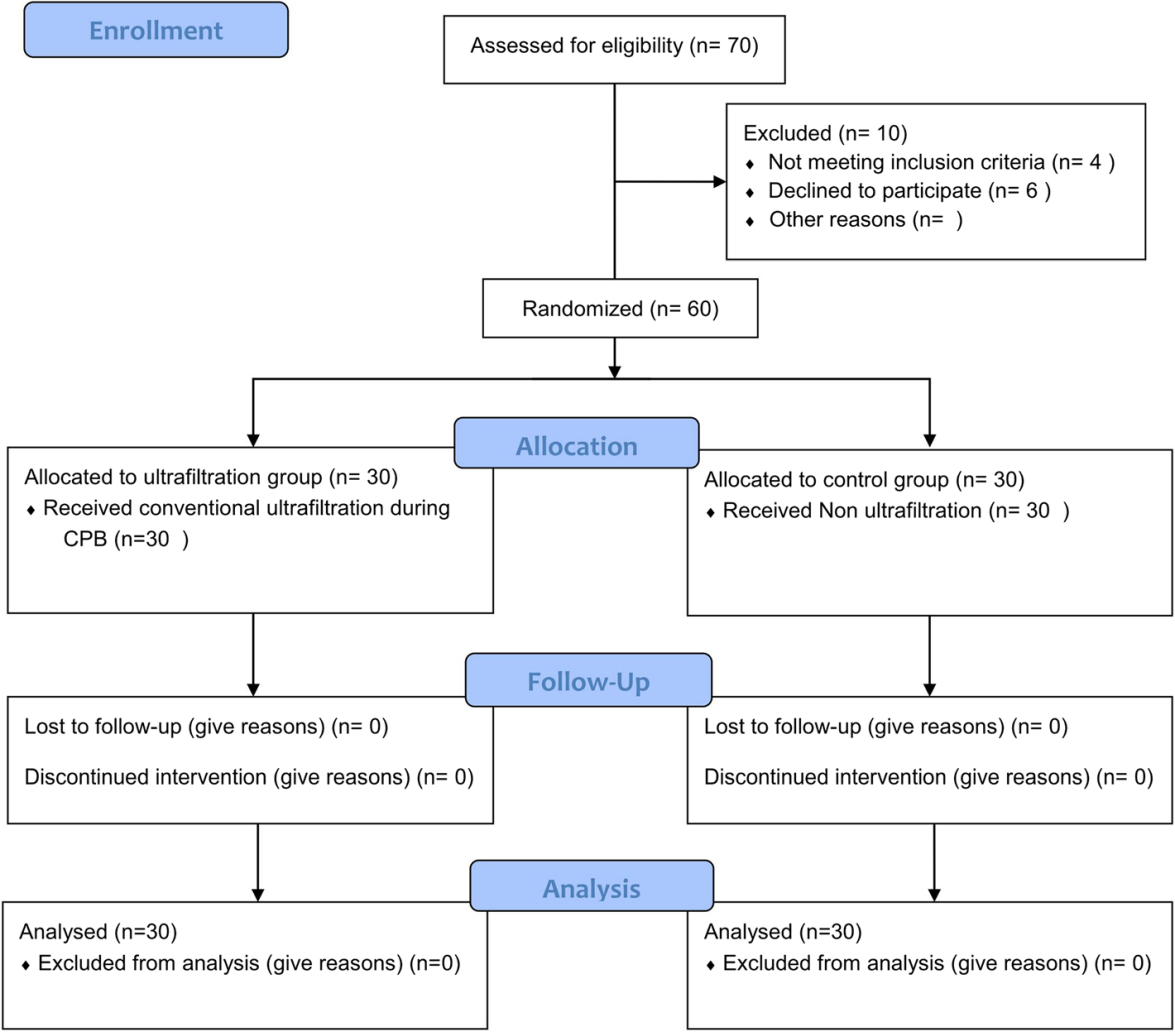
CONSORT flow diagram showing the number of patients at each phase of the study

Seventy participants aged four to 48 months were enrolled in the study. Four patients did not fulfil the inclusion criteria. Six patients refused to participate, and 60 patients completed the study (Fig. 1). Patients were assigned randomly into 2 groups: an ultrafiltration group (*n* = 30, received ultrafiltration during CPB) and a non-filtration (control) group (n = 30). The patients’ and clinical characteristics are presented in Tables 1 and 2. The patients underwent different types of congenital heart surgeries (Table 3).

**Table 1:** Patient characteristics; value presented as the mean (SD) and numbers

	Ultrafiltration group (*N* = 30)	Non-filtration group (*N* = 30)	*P* value
Age (months)	15.5 ± 14	19 ± 14.8	0.35
Sex (male/female)	(12/18)	(14/16)	0.30
Weight (kg)	8.04 ± 4.05	9.2 ± 3.7	0.25
Height (cm)	72.4 ± 21.2	78.5 ± 24.1	0.3

*N* Number of patients

**Table 2:** Duration of surgery, bypass duration, ventilation duration, ICU stay, ultrafiltration volume and volume of intraoperative fluid and blood; values are presented as the mean (SD) and numbers

	Ultrafiltration group (N = 30)	Non-filtration group (N = 30)	*P* value
Surgery duration (hours)	4.5 ± 0.6	4.5 ± 0.8	1
Bypass duration (min)	87.6 ± 33.7	78 ± 34.3	0.27
Ventilation days	1.04 ± 1.08	1.07 ± 1.2	0.91
ICU stay (days)	3.2 ± 1.3	3.3 ± 1.4	0.77
Ultrafiltration volume(mL)	580 ± 362.3		
Intraoperative fluid (mL)	283.1 ± 98.6	272 ± 127	0.71
Intraoperative blood (mL)	173.1 ± 44.9	199 ± 67.2	0.085

*N* number of patients, *ICU* intensive care unit

**Table 3:** Type and number of operations

Operations	Ultrafiltration group (*N* = 30)	Non-filtration group (*N* = 30)
VSD closure	22	18
ASD, VSD closure	1	2
Complete AV canal repair	5	9
Transitional AV canal repair	2	1

*VSD* ventricular septal defect, *ASD* atrial septal defect, *AV* atrioventricular

**Fig. 2:**
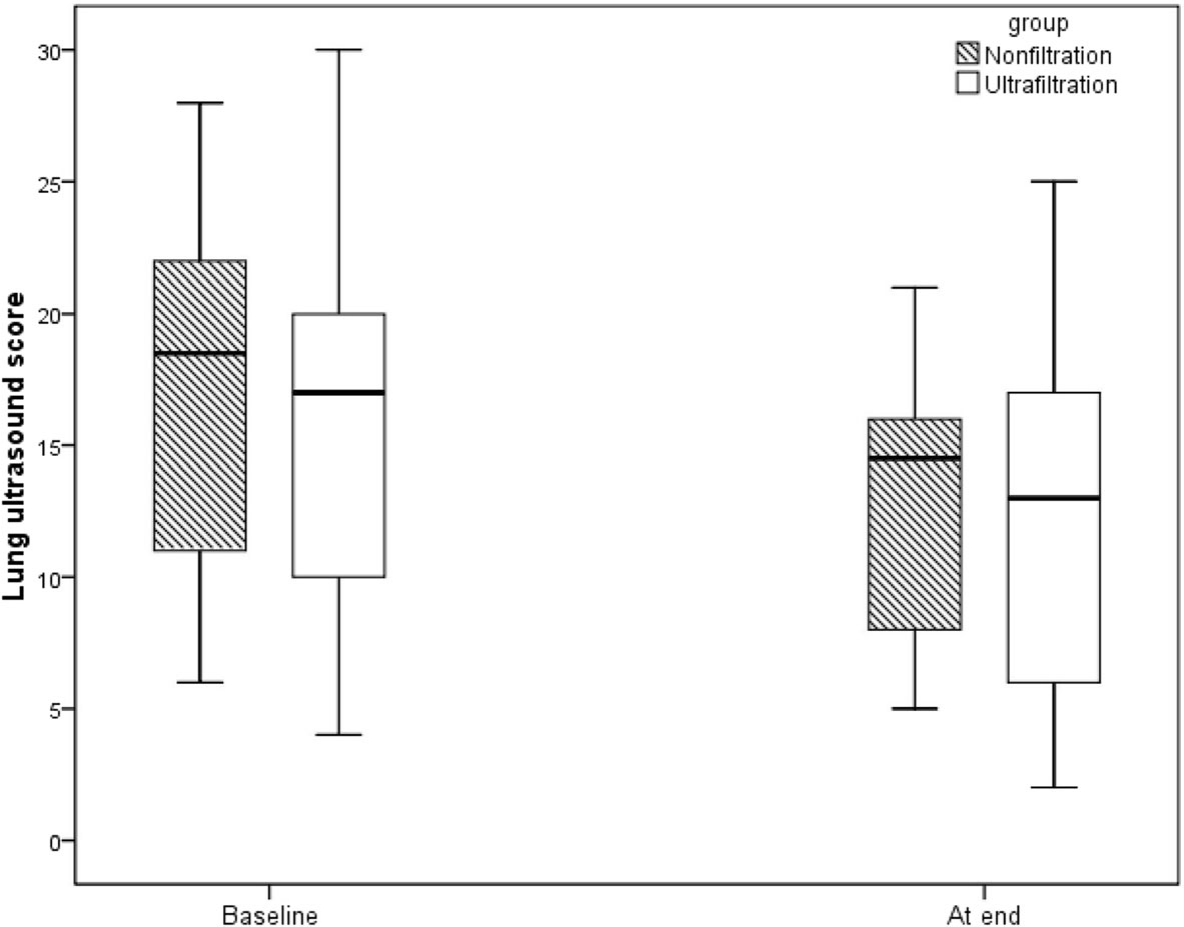
LUS score at baseline and at the end of surgery. Values are expressed as the median and IQR

The median and IQR of LUS scores in the ultrafiltration and non-filtration groups were assessed at baseline (17 (10–24) vs 18.5 (10.5–22.5)) and at the end of surgery (13 (6–18) vs 14.5 (7.5–17)). Relative to the baseline, the paired analysis revealed that lung scores at the end of the surgery were significantly lower, indicating an improvement of lung scores in both the ultrafiltration (*p* = 0.01) and non-ultrafiltration groups (*p* = 0.02). However, the LUS scores were comparable between the two groups both at baseline (*p* = 0.92) and at the end of surgery (*p* = 0.95) (Fig. 2).

**Fig. 3:**
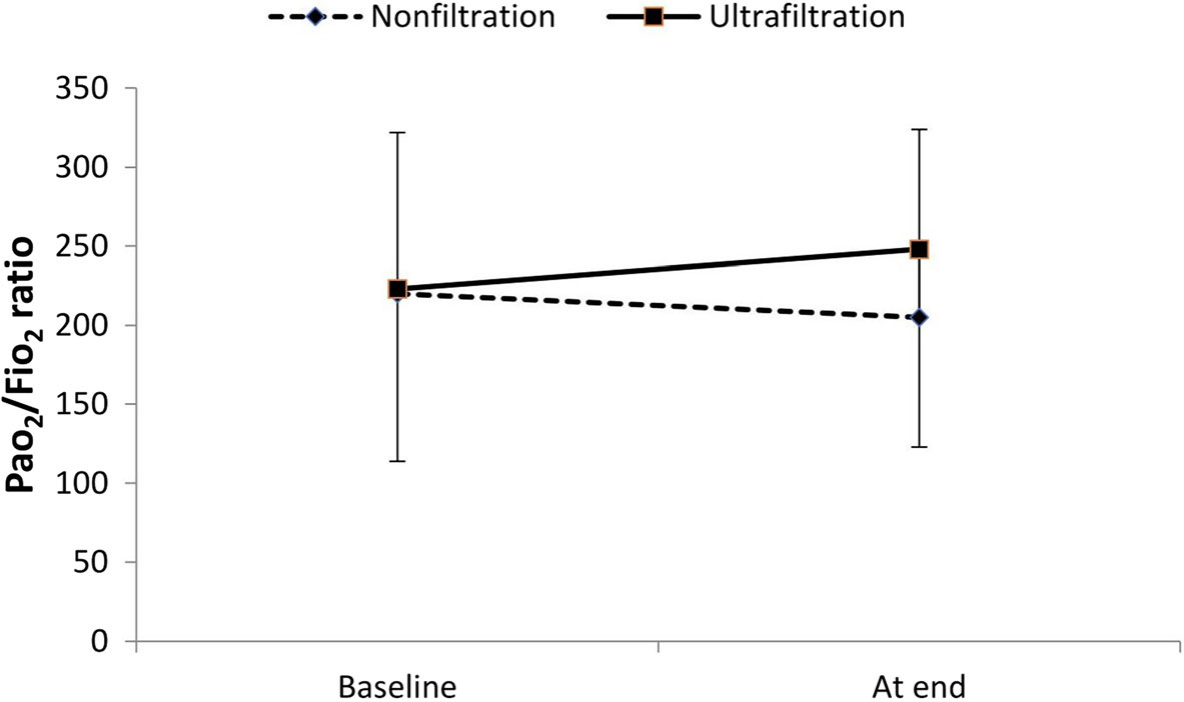
PaO2/FiO2 ratio at baseline and at the end of surgery. Values are presented as the mean (SD)

The baseline PaO2/FiO2 ratio was comparable between the ultrafiltration and non-filtration groups at baseline (220 ± 102 vs 205 ± 119) and at the end of surgery (223 ± 109 vs 248 ± 125). The PaO2/FiO2 ratio increased at the end of surgery in the ultrafiltration group compared to that in the non-ultrafiltration group, but there was no significant difference (*p* = 0.16) (Fig. 3). Within each group, the P/F ratio remained comparable.

**Fig. 4:**
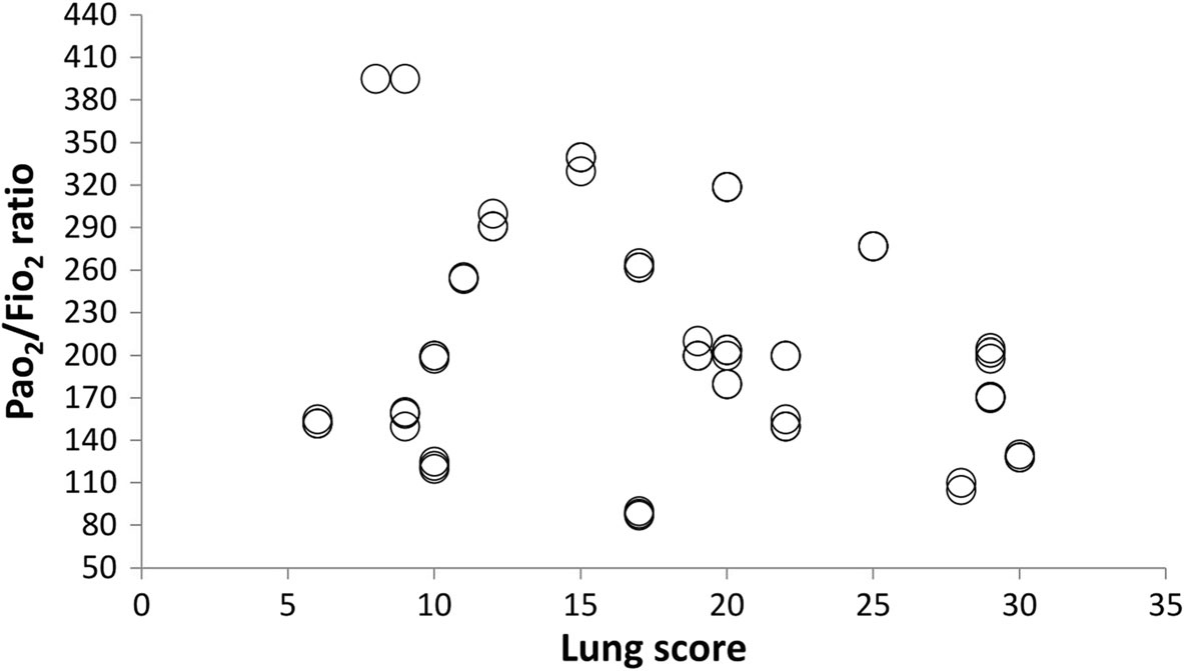
Correlation between the PaO2/FiO2 ratio and LUS score at baseline

**Fig. 5:**
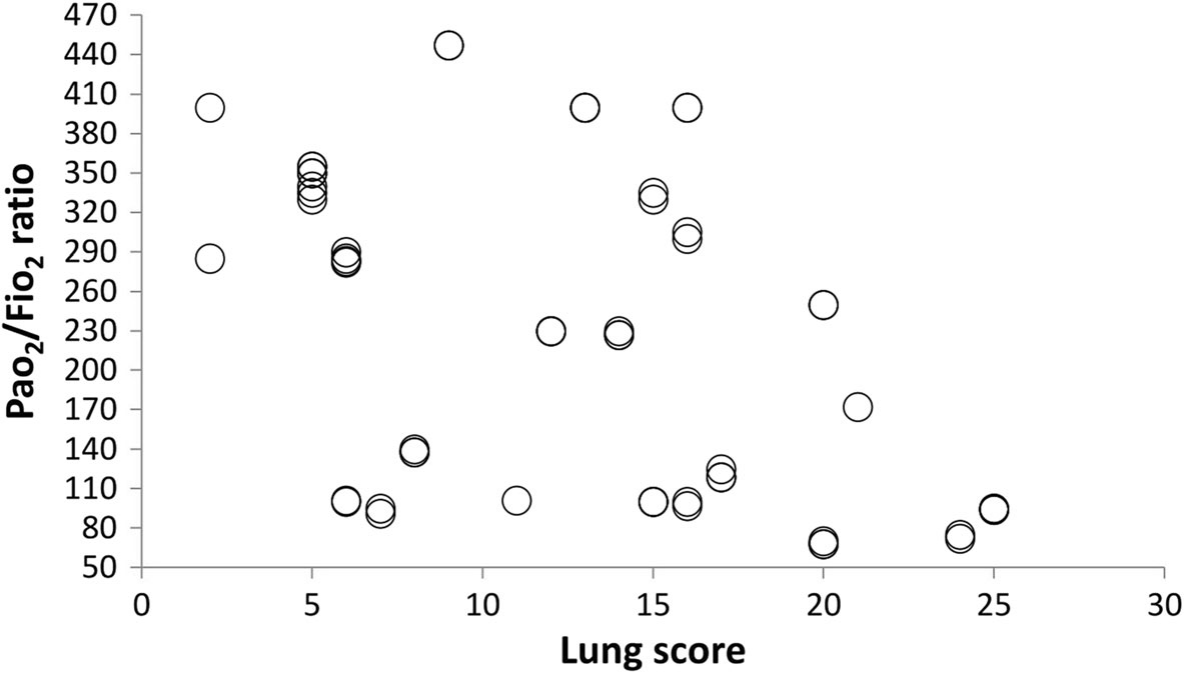
Correlation between the PaO2/FiO2 ratio and LUS score at the end of surgery

There was no correlation between the PaO2/FiO2 ratio and LUS scores at baseline readings (r = − 0.21, *p* = 0.31) (Fig. 4). At the end of surgery, a negative correlation was illustrated (r = − 0.41, *p* = 0.045) (Fig. 5). Heart rate (HR), systolic blood pressure (SBP), and diastolic blood pressure (DBP) were comparable both within and between groups at both time points (Table 4).

**Table 4:** Mean (SD) of the haemodynamic parameters

	Ultrafiltration group (*N* = 30)	Non-filtration group (*N* = 30)	*P* value
HR			
Baseline	139.7 ± 14	132 ± 11.5	0.18
At the end	150 ± 17.4	140 ± 14.5	0.14
SBP			
Baseline	97.3 ± 15.8	95.7 ± 17.5	0.82
At the end	90.5 ± 11.8	90.4 ± 15.2	0.98
DBP			
Baseline	55.6 ± 15.2	56.3 ± 11.4	0.91
At the end	58.3 ± 10.4	55.1 ± 9.7	0.44

*HR* heart rate, *SBP* systolic blood pressure, *DBP* diastolic blood pressure

Between groups, urea and creatinine were comparable at baseline (ultrafiltration vs non-filtration; 18.5 ± 5.2 vs 18.6 ± 6, *p* = 0.99; 0.55 ± 0.2 vs 0.62 ± 0.26, *p* = 0.13, respectively) and remained comparable at D1 postoperatively (29.6 ± 9 vs 27.2 ± 7.2, *p* = 0.49; 0.82 ± 0.25 vs 0.95 ± 0.15, *p* = 0.18, respectively)**.** Within each group, both urea and creatinine significantly increased at D1 compared to baseline values. (urea; *p* < 0.001), (create; *p* < 0.001).

Intraoperatively, urine output was comparable between the ultrafiltration group (328 ± 221 mL) and the non-filtration group (422 ± 236 mL, *p* = 0.11) and remained so at D1, being 321 ± 175 mL in the ultrafiltration group and 400 ± 145 mL in the non-filtration group, with *p* = 0.06.

Finally, both groups were comparable regarding the incidence of postoperative complications. Two patients in the non-filtration group developed mild lung congestion defined by increased bilateral basal crepitations with increased broncho-vascular markings on chest X-ray. Two patients in the ultrafiltration group developed mild chest infection diagnosed by increased chest secretions with a change in sputum colour and fever up to 38.5 °C. All 60 patients were discharged from the hospital without recorded mortality.

## Discussion

In the current study, the lung U/S score showed no significant difference between the ultrafiltration and non-filtration groups despite the improvement in the scores at the end of surgery compared to those at the beginning of surgery in both groups. Additionally, in the filtration group, the PaO2/FiO2 ratio insignificantly increased at the end of surgery compared to patients who did not undergo filtration on CPB.

In a study by Mallamaci et al. [14], LUS was used to detect pulmonary congestion in haemodialysis patients through the detection of “lung comets”, and it was found that standard ultrafiltration dialysis markedly reduced lung water and improved left ventricular performance in most cases. Similarly, Trezzi et al. [15] found a significant reduction in pulmonary B-lines following complete haemodialysis in patients on chronic dialysis, demonstrating the removal of volume overload. This reduction is significantly connected to the weight loss encountered during dialysis, emphasising a direct relationship between pulmonary B-lines and fluid balance.

Atelectasis caused by interstitial lung oedema remains the most significant effect of CPB on the lungs, inducing intravascular micro-aggregates to cause a decrease in PO_2_ (partial oxygen tension) and the PaO2/FiO2 ratio and an increase in the intrapulmonary shunt after bypass [1]. This complication is known as earlier postperfusion lung syndrome, in which microaggregates, damage of blood cells and leukocyte activation are aggravated by preoperative pulmonary hypertension in paediatric patients with ventricular septal defects or a complete AV canal [16].

Huang et al. [17] showed that in paediatric patients the combined use of balanced and modified ultrafiltration could effectively increase the concentration of the blood, alter the increase in detrimental inflammatory mediators, attenuate the lung oedema and inflammatory pulmonary injury together with mitigation of the pulmonary function impairment. The Huang explanation was that conventional ultrafiltration is a useful mean for decreasing fluid accumulation in the lungs, but it is not a satisfactory method in the paediatric population because of the lower volume in the venous reservoir. The concern in children is that the priming volume dilutes the patients’ blood content 2 to 3 times due to their small circulating volume, whereas it only equals 1/3 to 1/4 of the total blood volume in the adult population. This dilution can be reduced by decreasing the number of crystalloids and adding blood to the priming fluid, by using diuretics or by using ultrafiltration. Conventional ultrafiltration is more commonly applied during CPB, aiming for haemoconcentration. However, modified ultrafiltration is applied *after CPB* to remove inflammatory mediators such as IL6 and C3a [18].

Modified ultrafiltration was introduced to paediatric cardiac surgery because conventional ultrafiltration inadequately decreased the accumulated total body water and was less effective at removing inflammatory mediators. Two recent studies [19, 20] showed that the advantages of modified ultrafiltration over conventional ultrafiltration are only applicable to the immediate post-bypass period but not to the postoperative outcome parameters. In the last decade, very few studies have revealed the benefits of ultrafiltration in paediatrics because priming with blood increases the HCT and the ultrafiltration rate is inversely proportional to HCT. In the early 1990s, the protocol was an HCT of 24% for CPB, but now, perfusionists employ an HCT of 28% or even more for CPB. This difference in the HCT makes the use of ultrafiltration less efficient [21]. Thus, it might be wiser to control haemodilution earlier by minimizing crystalloids and priming with blood than to control the haemodilution at a later phase.

The improvement of the lung score at the end of the operation compared to the beginning of the operation could be attributed to the manual ventilation with high inspiratory pressure at the end of the bypass to recruit collapsed lungs. Another main factor for improvement may be CHD repair and the elimination of pulmonary circulation overload and lung congestion. The use of diuretics after bypass may have also decreased plasma water in the alveolar interstitial space, thereby increasing pulmonary compliance and improving gas exchange across the respiratory membrane, which in turn might have been responsible for the improved PaO2/FiO2 ratio and the lung score at the end of operation compared to those at baseline.

Naik et al. [22] postulated that the inefficiency of conventional ultrafiltration in reducing the total body water after CPB in paediatric patients occurs because the minimum prime volume is used in paediatrics. As per Naik et al. [22], the actual level in the reservoir remains very close to the alarm level, and thus, any filtration during bypass decreases the volume of the total circuit (patient and prime), resulting in the reduction of the actual fluid level in the venous reservoir already close to the alarm level. This means that more fluid must be added to the circuit, thereby negating the potential effects of ultrafiltration. Although no pulmonary data were measured in their study, this theory could explain the comparable lung scores at the end of the operation in the current study groups.

The use of ultrasound for diagnosing pleural and lung parenchyma abnormalities has increased in recent years [23]. LUS can be easily used to assess interstitial lung syndrome with increased EVLW. Increased EVLW may be diffuse (as in pulmonary oedema and adult respiratory distress syndrome [ARDS]) or focal (as lung contusion or pneumonia) [24]. LUS can also predict the volume and severity of EVLW, as there is a linear relationship between lung water and the number of B-lines. Increasing lung water was initially represented by B-lines, then as “white lung” formed by coalescent B-lines, and ultimately as alveolar consolidation [25]. Many studies [26–29] have demonstrated the high sensitivity and accuracy of LUS in diagnosing different pathologies, such as pneumothorax, pleural effusion, pneumonia and pulmonary embolism.

Urea and creatinine levels were comparable between groups in our study and remained so for the first postoperative day. In a study comparing conventional and modified ultrafiltration by Williams et al. [7], there was no significant difference in urea or creatinine levels between groups up to 48 h postoperatively. Increased urea and creatinine levels at D1 compared to baseline values in both groups might be explained by the combined effect of fluid administration and diuretic use postoperatively in the ICU.

In the current study, the PaO2/FiO2 ratio was negatively correlated with LUS scores, which agrees with the findings of Konstantinos Stefanidis et al. [30], who reported that transthoracic lung sonography could detect non-aerated lung area changes during a PEEP trial of patients with early ARDS. The non-ventilated regions in the dependent lung areas were significantly reduced when the PEEP increased from 5 to 10 to 15 cmH_2_O. These changes produced a significant increase in arterial oxygen partial pressure (74 ± 15 mmHg to 90 ± 19 mmHg to 102 ± 26 mmHg; *P* < 0.001, respectively).

No significant difference in haemodynamics was observed in either group due to the deliberate use of inotropes and vasoactive drugs to maintain haemodynamic stability throughout the study period.

Our study has some limitations; ideally, lung scores and PaO2/FiO2 ratios should have been recorded just before the start and at the end of bypass, but in the operating theatre environment, it would be impractical to apply ultrasound probes at these times. Further studies are required to assess the effect of modified ultrafiltration on the LUS score and on oxygenation.

## Conclusion

Conventional ultrafiltration did not alter EVLW when assessed by LUS and the oxygenation state. Ultrafiltration did not affect the urea and creatinine levels, length of ICU stay, number of ventilation days or mortality.

## Data Availability

Data are available from the authors (through contacting corresponding author) upon reasonable request after permission from Cairo University.

## Abbreviations

ACT: Activated clotting time
ARDS: Adult respiratory distress syndrome
AV: Atrioventricular
CHD: Congenital heart disease
CPB: Cardiopulmonary bypass
EVLW: Extravascular lung water
ICU: Intensive care unit
LUS: Lung ultrasound score
PaCO2: Carbon dioxide pressure
PaO2: Arterial oxygen pressure
Pao2/Fio2 Ratio: Arterial oxygen tension divided by the fraction of inspired oxygen
PEEP: Positive end-expiratory pressure
